# Editorial: Translational medicine in the diagnosis and treatment of cancer based on oncogenetics: from bench to bedside

**DOI:** 10.3389/fgene.2023.1210094

**Published:** 2023-11-13

**Authors:** Rui Cao, Ying Han, Changjing Cai, Jiao Hu, Changsheng Xing

**Affiliations:** ^1^ Department of Urology, Beijing Friendship Hospital, Capital Medical University, Beijing, China; ^2^ Department of Oncology, Xiangya Hospital, Central South University, Changsha, China; ^3^ National Clinical Research Center for Geriatric Disorders, Xiangya Hospital, Central South University, Changsha, China; ^4^ Department of Urology, Xiangya Hospital, Xiangya School of Medicine, Central South University, Changsha, China; ^5^ Department of Medicine, Keck School of Medicine, University of Southern California, Los Angeles, CA, United States

**Keywords:** translation medicine, bench to bedside, diagnosis, treatments, cancer

Cancer is a major public health problem worldwide. Clarifying cancer etiology and pathogenesis is of great significance to its prevention, diagnosis, and treatment. The concept of translational medicine, which was first mentioned as “bench to bedside” (B to B) pattern by Choi (1992), can be a useful method for cancer-related studies. Thus, in this Research Topic, we aim to highlight the emerging role of onco-genetics in cancer and discuss the potential challenges of cancer diagnoses and treatments, from the bench to the bedside.

In this Research Topic, Xiao et al. demonstrated the involvement of HOXC-4 in pan-cancer. They investigated the relationship between the expression level of HOXC-4 and prognosis, and additionally, the correlation between HOXC4 and clinical outcome of immunotherapy, together with anti-cancer drug sensitivity. This research provided a new potential biomarker in clinical process and shed new light on the anti-tumor immunotherapy (Xiao et al.).

Furthermore, the research of Cheng et al. indicated the advanced lung cancer inflammation index (ALI), which was a powerful prognostic and predictive biomarker in multiple myeloma (MM) patients and maintained the function of predicting the chemotherapy efficacy in MM patients receiving induction therapy. AMI was a promising biomarker in predicting the clinical outcome of immune checkpoint inhibitor efficacy in advanced non-small-cell lung cancer (Mountzios et al., 2021). In colorectal cancer patients, the preoperative ALI acted as a role in predicting the compositions and recurrence after curative resection (Horino et al., 2022). ALI also takes part in predicting the elderly patients with heart failure, which serves as an effective nutritional biomarker to predict the patient with better prognosis with higher ALI (Yuan et al., 2022). ALI exerted prognosis value in various kinds of cancers and immune therapy responses, together with heart disease. In the future, ALI may provide new therapy for cancer or cardiovascular diseases in the aspect of nutrition.

Neoadjuvant chemotherapy (NAC) is the standard treatment for muscle-invasive bladder cancer (MIBC), while the response of the NAC is still unclear for part of the MIBC patients (Hermans et al., 2018). The research by Li et al. constructed a signature to predict the prognosis of the patient and the clinical outcome and treatment of NAC with the help of bioinformatic analysis. The five-gene-based risk score predicted the NAC response with high accuracy, which might provide individualized treatment for each of different stage or pathological types of patients with more dawn.

RNA modification writer is widespread on all nucleotides and the modification is at the RNA level (Chen et al., 2021a). RNA modifying proteins may serve as a series of antitumor targets for drug discovery. The process of tumor formation also includes epi-transcriptome, which has been a hot Research Topic recently (He et al., 2019). The research of Cheng and Yi established a new signature which was associated with the distinct RNA modification clusters to test and verify the relationship between the signature and the related biological process, which was related to the tumor signaling pathway, tumor microenvironment, and clinical outcome. This research has provided new insight into the early diagnoses and treatment of cancer.

Ferroptosis is an advanced type of programmed cell death and is intensively connected to maintaining homeostasis and the development of detrimental diseases, especially tumorigenesis (Chen et al., 2021b). In recent years, researchers have found the role of lipid and mitochondria metabolism regulates ferroptosis in head and neck cancer drug-resistant (Wu et al., 2020). In the research of our Research Topic, Wei et al.conclude 47 genes related to ferroptosis and obtain the relation to the prognosis as well as immune signatures of head and neck squamous cancer (HNSCC) patients. The authors also assessed their conclusion in different clinical cohorts constructed by the FPRS scoring system. Thus, it provides a new prediction target and a therapeutic implication in clinical practice. Meanwhile, Yao et al. found that another cell death type, cuproptosis, has a close relationship with the prognosis of kidney renal clear cell carcinoma.

Interferon-γ (IFN-γ) plays a critical role in signaling pathway alterations in cancers (Jorgovanovic et al., 2020). At present, researchers have reported the inhibition of ER stress obviously prevents IFN-γ-triggered apoptosis, causing IFN-γ-mediated anticancer responses in lung cancers (Fang et al., 2021). As for Deng et al. research, an IFN-γ-related signature was found for its remarkable roles in the regulation of tumor progression, immune response, mutation, and tumor microenvironment in bladder cancer. The enhanced understanding of the IFN-γ-related signature would be expected to apply in clinic in the near future and be validated in more clinical cohorts.

Alkaline ceramidase 2 (ACER2) is an enzyme located in the Golgi complex participating in sphingolipid metabolizing, which is upregulated in most types of cancer (Xu et al., 2018). Liu et al. revealed that the expression level of ACER2 was correlated with bladder cancer in various aspects. Tumor microenvironment and immune checkpoint were hot research topics in cancer that correlate with ACER2 expression level significantly. Furthermore, ACER2 predicted the molecular subtypes to further estimate the immunotherapy response obliquely. Additionally, the ACER2 also obtained the ability to predict the clinical outcome of chemotherapy and immune therapy directly and accurately. The study also revealed that the ACER2 is significantly upregulated in other types of cancers, which may provide new insight into cancer treatment or new cancer biomarkers in the future.

In bone vascular endothelial cells, the immunolabeling of PECAM1 was revealed to show the vascular network (Wang et al., 2019). However, the tumor premetastatic environment can be formed before dissemination due to the tumor-derived factors released from primary tumors, including several growth factors associated with vascular networks (Wang et al., 2020). Thus we should attach importance to PECAM1 and bone metastases. In the research of Liang et al., they analyze the association and find that PECAM1 could be recognized as the potential biomarker that signifies the diagnosis and treatment of bone metastases and verified in bone metastatic tumor tissues. But there is also a limitation because the tissues in need were difficult to harvest. Thus, the authors believed the enlargement of the sample size was necessary and would continue collecting as many tissue samples as possible.

The PTPN family genes are promising prognostic biomarkers and therapeutic targets for various cancers. But the distinct roles of the PTPN family in acute myeloid leukemia (AML) have not yet been explored entirely. Liu et al. performed a comprehensive analysis which focused on the PTPN family expression profile and prognostic significance in AML. They screened out several differentially expressed PTPN family genes in AML. Especially, PTPN6 was one of these members and may be used as an AML diagnostic and prognostic marker (Liu et al.).

ZNF281 (zinc finger protein 281) plays critical roles in regulating embryonic stem cell (ESC) differentiation and maintaining cellular stemness. Hou et al. performed a pan-cancer analysis and indicated that ZNF281 was overexpressed in various cancers and related to shorter survival. Then, they correlated the ZNF281 with immune characters in tumor microenvironment. Overall, they provided new insights into the dual role of ZNF281 and approved that it was a potential biomarker for regeneration and tumor prognosis. Similarly, Guo et al. performed a pan-cancer analysis focused on Methyl methanesulfonate-sensitivity protein 22-like (MMS22L). They revealed the essential role of MMS22L as a tumor-regulating gene in human cancers while further emphasizing its feasibility as a novel molecular marker in hepatocellular carcinoma. Their findings provide an essential reference for the study of MMS22L in tumors. Meanwhile, Bao et al. found that overexpression of SPAG6 and low expression of NM23 are negatively related to the pathological grade, metastasis, and enneking stage and prognosis of osteosarcoma patients. This suggested that SPAG6 and NM23 should be considered candidate prognostic biomarkers for patients with osteosarcoma. Tang et al. confirmed FN1 is the potential novel biomarker for predicting poor prognosis and radio-resistance in HNSCC patients. Overexpression of FN1 plays an important role in the tumorigenesis, prognosis, and radio-resistance of HNSCC.

Taking advantage of novel computational approaches, publicly available datasets can be utilized to provide new scientific hypotheses. In this Research Topic, Ma and Yu identified the ADAM family as novel pan-cancer biomarkers for patient prognosis and potential clinical responses, using bioinformatics analyses and experimental approaches. The authors comprehensively analyzed the differential expression levels of ADAMs in multiple types of cancers, and explored the correlations between ADAMs and tumor microenvironment, stemness, immune subtype, and sensitivities to chemotherapy and immunotherapy. Since cancer immunotherapy shows impressive efficacy but limited responses in majority of cancer patients, the identification of new therapeutic targets will surely benefit the therapeutic decision-making and prediction of clinical outcomes.

In conclusion, it is our great honor to successfully recruit and attract numerous researchers to this Research Topic, who have contributed plenty of advanced, fascinating, and important works. However, limited by the present situation, we can only publish part of these articles which we believe are more applicable to our Research Topic. We believe with the progression of “B to B” studies, cancer treatments will be enormously improved, and eventually, future cancer patients will benefit from these research works.

## References

[B1] Chen H. Yao J. Bao R. Dong Y. Zhang T. Du Y. (2021a). Cross-talk of four types of RNA modification writers defines tumor microenvironment and pharmacogenomic landscape in colorectal cancer. Mol. Cancer 20, 29. 10.1186/s12943-021-01322-w 33557837 PMC7869236

[B2] Chen X. Kang R. Kroemer G. Tang D. (2021b). Broadening horizons: the role of ferroptosis in cancer. Nat. Rev. Clin. Oncol. 18, 280–296. 10.1038/s41571-020-00462-0 33514910

[B3] Choi D. W. (1992). Bench to bedside: the glutamate connection. Science 258, 241–243. 10.1126/science.1357748 1357748

[B4] Fang C. Weng T. Hu S. Yuan Z. Xiong H. Huang B. (2021). IFN-γ-induced ER stress impairs autophagy and triggers apoptosis in lung cancer cells. Oncoimmunology 10, 1962591. 10.1080/2162402X.2021.1962591 34408924 PMC8366549

[B5] He L. Li H. Wu A. Peng Y. Shu G. Yin G. (2019). Functions of N6-methyladenosine and its role in cancer. Mol. Cancer 18, 176. 10.1186/s12943-019-1109-9 31801551 PMC6892141

[B6] Hermans T. J. N. Voskuilen C. S. Van Der Heijden M. S. Schmitz-Dräger B. J. Kassouf W. Seiler R. (2018). Neoadjuvant treatment for muscle-invasive bladder cancer: the past, the present, and the future. Urol. Oncol. 36, 413–422. 10.1016/j.urolonc.2017.10.014 29128420

[B7] Horino T. Tokunaga R. Miyamoto Y. Hiyoshi Y. Akiyama T. Daitoku N. (2022). The advanced lung cancer inflammation index is a novel independent prognosticator in colorectal cancer patients after curative resection. Ann. Gastroenterol. Surg. 6, 83–91. 10.1002/ags3.12499 35106418 PMC8786697

[B8] Jorgovanovic D. Song M. Wang L. Zhang Y. (2020). Roles of IFN-γ in tumor progression and regression: a review. Biomark. Res. 8, 49. 10.1186/s40364-020-00228-x 33005420 PMC7526126

[B9] Mountzios G. Samantas E. Senghas K. Zervas E. Krisam J. Samitas K. (2021). Association of the advanced lung cancer inflammation index (ALI) with immune checkpoint inhibitor efficacy in patients with advanced non-small-cell lung cancer. ESMO Open 6, 100254. 10.1016/j.esmoop.2021.100254 34481329 PMC8417333

[B10] Wang Q. Liu K. Yang L. Wang H. Yang J. (2019). BoneClear: whole-tissue immunolabeling of the intact mouse bones for 3D imaging of neural anatomy and pathology. Cell Res. 29, 870–872. 10.1038/s41422-019-0217-9 31444467 PMC6796898

[B11] Wang M. Xia F. Wei Y. Wei X. (2020). Molecular mechanisms and clinical management of cancer bone metastasis. Bone Res. 8, 30. 10.1038/s41413-020-00105-1 32793401 PMC7391760

[B12] Wu Y. Yu C. Luo M. Cen C. Qiu J. Zhang S. (2020). Ferroptosis in cancer treatment: another way to rome. Front. Oncol. 10, 571127. 10.3389/fonc.2020.571127 33102227 PMC7546896

[B13] Xu R. Garcia-Barros M. Wen S. Li F. Lin C. L. Hannun Y. A. (2018). Tumor suppressor p53 links ceramide metabolism to DNA damage response through alkaline ceramidase 2. Cell Death Differ. 25, 841–856. 10.1038/s41418-017-0018-y 29229990 PMC5943524

[B14] Yuan X. Huang B. Wang R. Tie H. Luo S. (2022). The prognostic value of advanced lung cancer inflammation index (ALI) in elderly patients with heart failure. Front. Cardiovasc Med. 9, 934551. 10.3389/fcvm.2022.934551 36440019 PMC9697177

